# Antenatal Testing Education: Assessing Health Education and Health Knowledge in a High-Risk Pregnant Population

**DOI:** 10.1055/a-2891-4451

**Published:** 2026-06-26

**Authors:** Jessica Shaker, Marina Feffer, Abigail Winder, Mirelle M. Dawoud, Jameela Media, Mary J. Murphy, Hannah Pope, Ann K. Lal

**Affiliations:** 1 23356 Loyola University Health SystemMaywoodIllinoisUnited States; 2 2456 Loyola University ChicagoChicagoIllinoisUnited States

**Keywords:** health knowledge, patient satisfaction, antenatal testing

## Abstract

**Objective** This study aimed to assess health knowledge and patient satisfaction in patients undergoing antenatal surveillance.

**Study Design** This is a cross-sectional study that invited patients >18 years old, English-speaking, presenting for antenatal testing via biophysical profile. Patients completed a survey consisting of questions about health knowledge, demographic information, and patient satisfaction.

**Results** A total of 219 pregnant patients completed the survey. The average score on the health knowledge survey was 71.5% (standard deviation [SD] = 16.5). About 25% replied that coming to the weekly ultrasounds was difficult for them, 4% considered the test invasive, and 7% considered the test stressful to their mental health. Approximately 89% stated they felt the doctors explained the reason for the weekly ultrasound, and 92% of respondents stated they enjoyed the weekly ultrasounds. Among patients who identified as Hispanic/Latino, total antenatal knowledge scores were lower (68%, SD = 17.0) than those who identified as non-Hispanic/Latino (76%, SD = 14.7), *p* -value < 0.001. As education level increased, the total score increased significantly ( *p* -value < 0.001). No significant difference in total score was seen between varying racial groups nor between varying provider types.

**Conclusion** We found potential modifiable areas for improving health knowledge due to the different ethnic and educational backgrounds. We found an overall high satisfaction rate among the patients.

## Background

Health literacy and knowledge play a critical role in health care. Health literacy is defined as “people’s knowledge, motivation, and competences to access, understand, appraise, and apply health information in order to make judgments and make decisions in everyday life concerning health care, disease prevention, and health promotion to maintain or improve quality of life during the life course.” In short, health literacy is the degree to which patients can understand and utilize information and resources to make health care decisions. 1 It represents a modifiable factor that can be targeted to improve overall health outcomes. Poor health literacy is associated with increased risk of adverse behaviors in pregnancy. 2 Poor health literacy also perpetuates health disparities and continues poor health outcomes. 3 Furthermore, poor health literacy is associated with more negative views of the health system, which could lead to further distrust and difficulty with compliance with medical recommendations. 4

Research surrounding general antenatal care demonstrates that health knowledge surrounding antenatal care is insufficient. In a study performed by Guler et al, two-thirds of women had insufficient health literacy surrounding their pregnancy. 5 Additionally, studies show that low socioeconomic status has higher statistical odds of having inadequate health literacy, representing a high-risk group that may benefit from additional resources or methods of education. 6 In addition, women with adequate health literacy have been shown to make more informed choices regarding prenatal testing, which ultimately leads to lower anxiety levels for the patient. Given the significant time requirement and rationale for antenatal surveillance in pregnancy, this is an important component of prenatal care and requires significant patient involvement to complete. Little is known about health literacy and its relationship to antenatal surveillance in pregnancy. Based on a search, no previous studies have reported on the health literacy of antenatal surveillance. In addition, there are no previous publications surrounding patient satisfaction regarding antenatal surveillance.

Our study’s aim was to assess patient health knowledge of antenatal surveillance, specifically the biophysical profile (BPP), as this is the antenatal surveillance strategy used by our academic, tertiary care center. We also assessed patient satisfaction regarding attendance at antenatal surveillance appointments. Our goal was to ensure that patients are being appropriately counseled regarding antenatal surveillance and assess the burden these frequent visits had on patients’ lives.

## Materials and Methods

Our investigation is a prospective survey collection tool used to assess patient health knowledge surrounding antenatal testing. The study was conducted at a large academic center. Surveys were given to all patients presenting to the maternal–fetal medicine (MFM) ultrasound unit for antenatal testing. At our center, all antenatal testing is performed with BPPs, and all antenatal testing for our patients is performed at one central unit, with MFM sonographers performing the ultrasounds and MFM physicians interpreting each ultrasound. The surveys were handed out from March 2023 to January 2024.

Institutional Review Board (IRB) approval was obtained (LU no.: 216729). Every patient presenting for antenatal testing was approached for study inclusion by study personnel. All patients were given a detailed letter about the study prior to participation. Once the letter was reviewed and patients agreed to participate, patients completed a health knowledge and patient satisfaction survey about antenatal testing. Study inclusion criteria included patients >18 years old, English-speaking, and undergoing antenatal testing at our institution. Exclusion criteria were <18 years old, non-English-speaking, undergoing antenatal testing for a nonrecurring indication, having already completed the survey, or planned delivery at an outside institution. This survey was a 17-question survey, including demographics, health knowledge, and patient satisfaction regarding antenatal testing. The survey was completed during their scheduled ultrasound appointment, and the approximate completion time was 5–10 min. Participants were not allowed to complete the survey at home and return it at subsequent visits. If patients declined participation, they were not asked at subsequent visits.

Survey information was collected and recorded in a secure database. Demographic information was collected through the survey as well as with the electronic medical record, including age, gravidity, parity, type of physician performing obstetric care, insurance status, body mass index (BMI), education level, and medical comorbidities. Demographic and birth characteristics of the women were collected via self-report, and demographic information was supplemented via chart review for missing information. Antenatal knowledge survey questions were designed to cover multiple levels of knowledge, including the name and purpose of the test, indications for antenatal testing, and implications of test results. Antenatal knowledge scores were computed as a percentage of questions answered correctly. Differences in overall test scores between race, ethnicity, and provider type were assessed via one-way analysis of variance (ANOVA) testing. A test for trend between total score and increasing educational level was assessed via a Wilcoxon-type test for trend. 7 Differences between groups on individual question responses were assessed via chi-square test or test for trend, where appropriate. Comparisons between groups did not include responses from respondents who selected “prefer not to answer” for the patient characteristic. All tests were two-sided and had a significance threshold of *p* < 0.05. 8

## Results

We identified a total of 284 pregnant patients who met the inclusion criteria, and 65 patients declined to participate. The demographic information of our study population is demonstrated in Table 1 . Of the 219 women surveyed, the average age was 32.9 years (standard deviation [SD] = 6.1) with an average pregravid BMI of 34.1 (SD = 7.7). Most women were White (53%), followed by Black or African American (19%), other race (17%), or preferred not to answer (11%). Just over half of the women surveyed identified as Hispanic or Latino (53%), followed by 45% non-Hispanic or Latino, and 2% preferred not to answer. Most women responded they had a college degree or higher (64%), followed by high school or General Educational Development (GED) or less (34%), and preferring not to answer (2%). We surveyed patient-reported household income, with 24% of women reporting their household income to be less than $50,000, 27% between $50,000 and 100,000, 22% between $100,000 and 200,000, 5% reported more than $200,000, and 22% selected “prefer not to answer.” Regarding their pregnancy provider, most women reported seeing a general obstetrician (47%), followed by a high-risk/MFM doctor (37%), midwife/nurse practitioner (11%), and 5% were unsure. Most deliveries were either normal spontaneous or vacuum-assisted vaginal deliveries (57%), followed by cesarean section (42%), with vacuum-assisted cesarean section (1%). Less than one-fifth of the deliveries required neonatal intensive care unit (NICU) admission (18%). Approximately 30% of patients reported that this was their first pregnancy. The two most common comorbid conditions were obesity (68%) and hypertension (44%).

**Table 1:** Patient characteristics.

Total ( *N* = 219)		Mean (SD)
Age ( *N* = 218)		32.9 (6.1)
Pregravid BMI		34.1 (7.7)
Number of prenatal visits ( *N* = 214)		10.5 (2.5)
		***N* (%) **
Race	White	117 (53.4)
	Black	41 (18.7)
	Other	38 (17.4)
	Prefer not to answer	23 (10.5)
Ethnicity	Yes	115 (52.5)
	No	99 (45.2)
	Prefer not to answer	5 (2.3)
Highest level of education completed ( *N* = 195)	Middle school	4 (2.1)
	High school/GED	62 (31.8)
	College	80 (41.0)
	Professional degree	45 (23.1)
	Prefer not to answer	4 (2.1)
Household income ($; *N* = 192)	<50,000	46 (24.0)
	50,000–100,000	51 (26.6)
	100,000–200,000	43 (22.4)
	>200,000	9 (4.7)
	Prefer not to answer	43 (22.4)
Who is your pregnancy doctor?	High-risk/MFM doctor	80 (36.5)
	OB doctor	102 (46.6)
	Midwife/Nurse practitioner	24 (11.0)
	Family practice doctor	2 (0.9)
	Not sure	11 (5.0)
Mode of delivery ( *N* = 216)	NSVD	119 (55.1)
	VAVD	5 (2.3)
	CS	91 (42.1)
	Other	1 (0.5)
NICU admission ( *N* = 201)		36 (17.9)
First pregnancy		65 (29.7)
Comorbid conditions	Obesity	148 (67.6)
	Hypertension	96 (43.8)
	T1DM	4 (1.8)
	T2DM	14 (6.4)
	GDMA	51 (23.3)
	Lupus	1 (0.5)
	Kidney disease	0 (0.0)
	Heart disease	0 (0.0)
	IUGR	20 (9.1)
	AMA	93 (42.5)
	Asthma	27 (12.3)
	Depression	24 (11.0)
	Anxiety	27 (12.3)
	Other	137 (62.6)

Abbreviations: AMA, advanced maternal age; BMI, body mass index; CS, cesarean delivery; GDMA, gestational diabetes on medication; GED, general educational development; IUGR, fetal growth restriction; MFM, maternal–fetal medicine; NICU, neonatal intensive care unit; NSVD, normal spontaneous vaginal delivery; SD, standard deviation; SVD, normal spontaneous vaginal delivery; T1DM, type 1 diabetes mellitus; T2DM, T1DM, type 2 diabetes mellitus; VAVD, vacuum assisted vaginal delivery.

Note: The entire study cohort, *n* = 219, demographic information, and pregnancy outcomes.

Among all respondents, the average percent correct on the antenatal knowledge quiz was 71.5% (SD = 16.5). Almost all respondents answered question no. 3 correctly (92%), while only 13% answered question no. 6 correctly. Following the knowledge-based questions, the survey included five questions regarding perceptions around antenatal testing. Many patients did not respond to these questions; the varying sample sizes are listed in Table 2 . Of those who responded, 25% replied that coming to the weekly ultrasounds was difficult for them, 4% considered the test invasive, and 7% considered the test stressful to their mental health.

**Table 2:** Antenatal testing knowledge-based survey responses among total sample health knowledge-based survey responses for the entire cohort, *n* = 219.

Knowledge-based survey responses	Mean (SD)
Total score	6.4 (1.5)
Percent correct	71.5 (16.5)
	***N* (%) **
Q1	183 (83.6)
Q2	176 (80.4)
Q3	202 (92.2)
Q4	176 (80.4)
Q5	173 (79.0)
Q6	30 (13.7)
Q7	122 (55.7)
Q8	163 (74.4%)
Q9	185 (84.5)
**Barriers to care**	***N* (%) **
Coming to weekly ultrasounds is difficult for me ( *N* = 191)	48 (25.1)
I consider this test invasive ( *N* = 181)	7 (3.9)
Coming to weekly ultrasounds is stressful for my mental health ( *N* = 188)	13 (6.9)
I feel like the doctors explained the reason for weekly ultrasounds ( *N* = 188)	168 (89.4)
I enjoy the weekly ultrasounds ( *N* = 182)	167 (91.8)

Abbreviation: SD, standard deviation.

However, 89% stated they felt the doctors explained the reason for the weekly ultrasounds, and 92% of respondents stated they enjoyed the weekly ultrasounds.

Among patients who identified as Hispanic or Latino, total antenatal knowledge scores were slightly lower (68%, SD = 17.0) than those who identified as non-Hispanic or Latino (76%, SD = 14.7), *p* -value <0.001 ( Table 3 ). Among varying educational levels, as education increased, total score increased significantly ( *p* -value < 0.001, Table 3 ). No significant difference in total score was seen between varying racial groups nor between varying provider types.

**Table 3:** Antenatal knowledge stratified by patient characteristics’ health knowledge-based survey responses by patient characteristics.

		Mean percent correct (SD)	*p* -Value ^a^
Race	White	73.7 (16.0)	0.15
	Black	73.2 (15.5)	
	Other	67.8 (17.5)	
Ethnicity	Hispanic	68.0 (17.)	<0.001
	Non-Hispanic	75.9 (14.7)	
Education level	High school/GED or less	66.7 (14.7)	<0.001 ^b^
	College	74.0 (14.7)	
	Professional	79.3 (15.6)	
Provider type	High-risk/MFM	69.6 (16.1)	0.220
	OB GYN	73.4 (17.1)	
	Midwife/Nurse	72.2 (13.9)	
	Not sure	64.6 (17.1)	

Abbreviations: GED, general educational development; MFM, maternal–fetal medicine; OB GYN, obstetrician-gynecologist; SD, standard deviation.

^a^ One-way analysis of variance (ANOVA).

^b^ Wilcoxon-type test for trend.

## Discussion

Antenatal testing can be a difficult-to-understand, onerous, and stressful aspect of prenatal care. Our study aimed to assess health knowledge and explore patient satisfaction surrounding antenatal testing in our patient population. Our results demonstrated that patients were able to answer, on average, 71.5% of health knowledge questions correctly on BPPs. When designing our survey, it was agreed that approximately 67%, or at least two-thirds of questions answered correctly, would correlate to a satisfactory score. Based on our results, it was determined that our patients had a proficient level of health knowledge.

Our study identified potential opportunities to improve health knowledge among patients of different ethnic and educational backgrounds. Our results identified a difference in mean percent correct between Hispanic or Latino and non-Hispanic or Latino populations (68.0 and 75.9% correct, respectively). Furthermore, we found a dependent relationship between increasing education level and increasing score, with high school GED, college, and professional degree, with 66.7, 74.0, and 79.3%, respectively. These disparities are critical in understanding how patients engage with and comprehend antenatal care information. Health care systems must consider these factors when developing strategies to improve health knowledge, particularly in marginalized communities. Tailored interventions that address the specific needs of these populations may help bridge the gap and ensure equitable access to prenatal care, as well as better patient understanding of prenatal care requirements.

One interesting aspect of our study was the overall positive experience patients reported in undergoing antenatal testing. The majority of patients enrolled in our study reported they enjoyed the weekly ultrasounds (92%) and did not find it difficult to attend. This was a surprising finding as antenatal testing confers frequent visits in addition to routine prenatal visits that can impose additional transportation and financial burdens, require additional time off work, necessitate childcare, and potentially add further stress to an already high-risk pregnancy. However, only 13 patients (6.9%) felt that the additional ultrasounds were stressful to their mental health. Furthermore, our patients also reported that they understood the reason for the ultrasound. This speaks of the excellent counseling by their treating provider and may offer a potential explanation for the patients’ overall positive experience.

Our study had several strengths. Our institution is a large, academic medical center that sees a variety of patients, making these results applicable to many different patient populations. We recruited 219 patients in total. In addition, the survey was administered to all patients who presented for antenatal testing. Our study assessed patient knowledge as well as patient demographics, allowing us to assess patient knowledge in relation to their demographics.

A limitation of our study was the lack of a previously validated assessment tool to assess health knowledge. We independently developed our assessment survey, which may not correlate with overall health knowledge. In addition, it is possible that the language of the survey was designed with implicit bias in the way the questions were phrased, which generated a difference in scores among people of different ethnic groups. Our survey did not include non-English-speaking patients, so their results cannot be assessed. We also acknowledge that we were not able to assess where information regarding the ultrasounds came from, as neither our providers nor ultrasound technicians had a script. However, given that there is a lack of established research in this area, it is our hope that this can provide a foundation for further research.

Our study aimed to offer insight into health knowledge surrounding antenatal testing, which can often be a confusing, burdensome, and anxiety-inducing aspect of pregnancy. Our study highlighted that for our patient population, attending weekly antenatal testing was not burdensome for the majority of patients. In addition, the majority of patients had overall high knowledge scores, with average scores of >70%. This shows that most of our patients had a good understanding of what antenatal testing is and an understanding of the purpose of antenatal testing. It also highlighted differences in ethnic groups, as well as educational levels, which could lead to improved counseling in certain patient groups.

Health knowledge levels can be tied to access to health care, engagement with their medical care, and patient outcomes. Literature surrounding health knowledge has shown it is associated with increased hospitalization, higher utilization of emergency medical services, and poorer ability to take medications properly. 9 10 Our study’s findings suggest that health knowledge surrounding antenatal testing at our institution is generally good. Health knowledge may be associated with different ethnicities or different education levels, which suggests that more education for certain groups could lead to better health knowledge. Development of further counseling tools or techniques may enhance health knowledge in these groups and narrow the gap among our patients. Our findings also suggest that patients overall felt antenatal testing was a positive experience, suggesting our providers have been successful in supporting and empowering their patients. Further research will be needed to create standardized tools to assess health knowledge and implement strategies to improve patients’ understanding of antenatal testing with the goal of ameliorating health disparities and improving perinatal outcomes.

## Conclusion

In an academic tertiary care center, overall patient health knowledge regarding antenatal testing was good. Patient satisfaction surrounding antenatal testing with BPPs is good. Patients with lower education levels and Hispanic or Latino ethnicity had lower health knowledge scores than patients with higher education levels and non-Hispanic or Latino ethnicity. This highlights an area for improvement in counseling about why antenatal testing is performed.

## Data Availability

The datasets used and/or analyzed during the current study are available from the corresponding author on reasonable request.
